# A Three-Dimensional Interlocked Heterojunction Photoanode for Sustainable Metal Corrosion Control in Marine Environments

**DOI:** 10.3390/nano16110652

**Published:** 2026-05-22

**Authors:** Xiaoyan Liu, Chuchu Chen, Yumei Zhang, Xilong Liu, Xiurui Zhang, Leiying Han

**Affiliations:** 1 College of Civil and Transportation Engineering, Hohai University, Nanjing 210098, China; liuxiaoyan@hhu.edu.cn (X.L.); 251604080028@hhu.edu.cn (X.Z.); 250204050001@hhu.edu.cn (L.H.); 2 Institute of Corrosion Protection, Hohai University, Nanjing 210098, China; 3 College of Materials Science and Engineering, Hohai University, Nanjing 213000, China; 231325010018@hhu.edu.cn (C.C.); 231625010017@hhu.edu.cn (X.L.)

**Keywords:** durability, three-dimensional interlocked architecture, heterojunction semiconductors, ternary sulfide, photoelectrochemical cathodic protection

## Abstract

The development of highly efficient and stable photoanodes is essential for advancing photoelectrochemical cathodic protection towards practical applications. Herein, a novel ternary sulfide heterojunction was engineered through the construction of a three-dimensional interlocked architecture of ZnIn_2_S_4_ on SnIn_4_S_8_ nanosheets via a sequential hydrothermal synthesis. This unique three-dimensional interlocked configuration creates an intimate interface and continuous charge transfer highways, effectively addressing the slow electron movement and poor interfacial contact that plague conventional photoelectrodes. Spectroscopic and electrochemical analyses verified the formation of a Type-II band alignment, which drives the directional migration of photogenerated electrons from ZnIn_2_S_4_ to SnIn_4_S_8_ under an intrinsic built-in electric field. Upon coupling with 304 stainless steel, the ZnIn_2_S_4_/SnIn_4_S_3_ heterojunction exhibited outstanding photoelectrochemical cathodic protection performance. It delivered impressive photocurrent densities of 15.22, 19.76, and 72.27 μA·cm^−2^ in 3.5 wt% NaCl, 0.1 M Na_2_S_2_O_3_, and 0.1 M Na_2_S/NaOH electrolytes, respectively, along with a prominent 720 mV cathodic potential shift in the Na_2_S/NaOH system. Most importantly, its good activity and stability in the scavenger-free 3.5 wt% NaCl solution and natural seawater highlight the strong practical potential of this 3D interlocked photoanode for sustainable marine metal corrosion control. Through a strategic multi-electrolyte assessment, the underlying protection mechanisms were decoupled, revealing that the synergy between the heterojunction-induced charge separation enabled by the three-dimensional interlocked structure and electrolyte-specific hole scavenging is key to the enhanced performance.

## 1. Introduction

Metal corrosion inflicts enormous economic losses worldwide on an annual basis [1,2,3], highlighting the imperative need for developing efficient and sustainable novel anti-corrosion technologies. In this context, photoelectrochemical cathodic protection (PECCP) presents an environmentally friendly solution, wherein semiconductor photoanodes are utilized to convert solar energy into protective current for metal corrosion prevention [4,5,6,7,8,9,10]. This approach aligns with the growing emphasis on green and sustainable technologies in chemical engineering. Conventional TiO_2_-based photoanodes suffer from inherent limitations, including a wide bandgap (E_g_ = 3.2 eV) that restricts light absorption to ultraviolet wavelengths (<387 nm), high charge carrier recombination rates, and poor sustained protection in dark conditions [11,12,13]. In recent years, narrow-bandgap metal sulfides (Eg < 2.5 eV) have emerged as promising candidates due to their superior visible-light harvesting capability (400–700 nm) [14,15]. However, binary sulfides (e.g., CdS, MoS_2_) are plagued by severe photocorrosion [16,17,18], necessitating the exploration of novel stable materials. Notably, binary/ternary or binary/binary sulfide heterostructures (e.g., CdS/ZnIn_2_S_4_ [19] or CdS/MoS_2_ [20]) can partially enhance photoresponse but fail to address the intrinsic photocorrosion of binary components or the charge recombination induced by band misalignment, thereby limiting their long-term stability in PECCP applications.

Ternary sulfide SnIn_4_S_8_, with an optimal bandgap (E_g_ ≈ 2.0–2.4 eV) and strong visible-light responsiveness, has demonstrated excellent performance in photocatalytic degradation [21,22] and heavy metal reduction [23,24]. Nevertheless, its practical application is hindered by poor photostability, insufficient hole oxidation capability, and interfacial charge transport resistance [25,26]. Constructing heterojunctions has been recognized as an effective strategy to mitigate these issues [9,27,28,29]. Meanwhile, another ternary sulfide, ZnIn_2_S_4_, exhibits tunable bandgaps (2.2–2.8 eV), a unique layered structure, and remarkable chemical stability [30,31,32], making it highly efficient in photocatalytic hydrogen evolution [33,34,35] and CO_2_ reduction [36,37]. Its two-dimensional nanosheet architecture not only provides abundant active sites but also facilitates charge separation via interlayer electric fields [38]. Therefore, coupling two ternary sulfides can synergistically mitigate photocorrosion while enabling precise band alignment due to their inherent lattice and band structure compatibility. Remarkably, the well-matched band structures of SnIn_4_S_8_ and ZnIn_2_S_4_ facilitate the formation of a built-in electric field at their interface, promoting charge separation and enhancing redox capability [39]. Further studies confirmed that such band alignment significantly reduced interfacial electron transfer barriers. Additionally, the nanoarray morphology of ZnIn_2_S_4_ increases active site density and optimizes interfacial contact uniformity, further improving charge separation efficiency [38]. Despite these advances in energy conversion, the adaptation of SnIn_4_S_8_ and ZnIn_2_S_4_ in PECCP—particularly in corrosive environments (e.g., Cl^−^, S^2−^, S_2_$O_{3}^{2-}$)—remains underexplored.

This study designed a three-dimensional (3D) interlocked ZnIn_2_S_4_/SnIn_4_S_8_ (ZIS/SIS) heterojunction photoanode via a solvothermal-hydrothermal two-step method to precisely regulate interfacial properties. The construction of this 3D interlocked architecture is imperative for sustainable corrosion control, as it fundamentally addresses the limited interfacial contact and high charge transfer resistance prevalent in conventional heterostructures, thereby ensuring efficient and durable electron supply for long-term cathodic protection in aggressive marine conditions. Compared to conventional physically mixed heterostructures, the 3D interlocked architecture provides continuous electron transport pathways and maximizes active interfacial area [40]. To evaluate its practical application potential for sustainable corrosion control in marine environments, this study conducted systematic investigations under different electrolytes (3.5 wt% NaCl, 0.1 M Na_2_S_2_O_3_, Na_2_S/NaOH, and natural seawater), revealing the anti-passivation mechanism and long-term stability of ZIS/SIS in chloride-rich environments. Furthermore, this comprehensive electrolyte comparison serves a dual purpose: firstly, to benchmark the material’s performance under idealized conditions (with scavengers) and push its limits; secondly, and more importantly, to rigorously validate its practical applicability and durability in realistic, scavenger-free marine environments (NaCl and seawater), which is the ultimate scenario for sustainable marine corrosion protection. This work elucidates the role of the highly matched lattice interface within the 3D interlocked framework in constructing efficient charge transfer pathways and deciphers the intrinsic mechanisms for the enhanced PECCP performance across diverse electrolytes.

## 2. Materials and Methods

### 2.1. Synthesis of SnIn_4_S_8_ Photoanode

The SnIn_4_S_8_ two-dimensional nanosheet arrays (NSAs) were hydrothermally grown on fluorine-doped tin oxide (FTO) glass substrates (15 × 10 × 2.2 mm) [41]. Prior to synthesis, the FTO, Luoyang Guluo Glass Co., Ltd., Luoyang, China, substrates were ultrasonically cleaned with isopropanol, Sinopharm Group Shanghai Chemical Reagents Co., Ltd., Shanghai, China, ethanol, Sinopharm Group Shanghai Chemical Reagents Co., Ltd., Shanghai, China, and deionized water, Zhongdong Chemical & Glass Co., Ltd., Nanjing, China (15 min each), then dried at 60 °C to ensure proper adhesion. For film growth, an ethanol-based precursor solution containing 2.5 mM SnCl_4_·5H_2_O, 10 mM InCl_3_·4H_2_O, and 25 mM thioacetamide (TAA), Sinopharm Group Shanghai Chemical Reagents Co., Ltd., Shanghai, China, was stirred for 20 min. Two pretreated FTO substrates were placed face-down at a 45° angle in a 20 mL Teflon-lined autoclave, which was then filled with the precursor solution. The hydrothermal reaction proceeded at 180 °C for 6 h. After cooling, the resulting yellow SnIn_4_S_8_ NSA films were rinsed with water and absolute ethanol, followed by drying at 60 °C for 4 h. All chemicals were of analytical grade and used without further purification.

### 2.2. Synthesis of ZnIn_2_S_4_/SnIn_4_S_8_ Heterojunction Photoanodes

**Figure 1:**
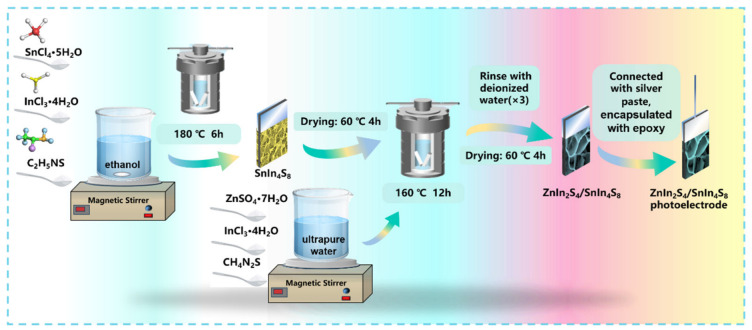
Schematic diagram of the fabrication process for ZnIn_2_S_4_/SnIn_4_S_8_ heterojunction film.

The ZnIn_2_S_4_/SnIn_4_S_8_ heterostructure was successfully fabricated on SnIn_4_S_8_ thin films via a hydrothermal deposition method [42], with the detailed preparation procedure illustrated in Figure 1. The experimental steps were performed as follows: First, 5 mM ZnSO_4_∙7H_2_O, 10 mM InCl_3_∙4H_2_O, and 20 mM C_2_H_5_NS were sequentially dissolved in ultrapure water under magnetic stirring for 30 min to obtain a homogeneous transparent precursor solution. Subsequently, two pre-prepared FTO-SnIn_4_S_8_ substrate films were placed in a 20 mL Teflon-lined autoclave at approximately 45° inclination angle with their conductive sides facing downward, followed by adding the precursor solution until complete substrate immersion. The sealed autoclave was then maintained at 160 °C for 12 h in a drying oven. After reaction, the obtained samples were repeatedly rinsed with deionized water to remove surface impurities and dried at 60 °C for 4 h, yielding yellow ZnIn_2_S_4_/SnIn_4_S_8_ nano-heterojunction films (denoted as ZIS/SIS). For comparison, pure ZnIn_2_S_4_ nanosheet films with pale yellow color were synthesized under identical hydrothermal conditions using blank FTO substrates. For photoelectrode fabrication, the polytetrafluoroethylene (PTFE) tape was removed from film samples to expose 10 mm × 5 mm conductive areas, which were then connected with copper wires using conductive silver paste before being encapsulated with epoxy AB glue. The photoelectrodes were obtained after complete solidification.

### 2.3. Characterization Techniques

#### 2.3.1. X-Ray Diffraction (XRD)

X-ray diffraction (XRD) was carried out on a Rigaku D/max-2200PC diffractometer, Rigaku Corporation, Tokyo, Japan, to identify the crystal phase structure of the samples. The scanning range was set from 10° to 80° at a scanning rate of 5°/min. Direct measurements were performed on the as-grown surface of thin-film samples and the washed/dried powder samples, respectively.

#### 2.3.2. Field-Emission Scanning Electron Microscopy (FE-SEM) and Energy-Dispersive X-Ray Spectroscopy (EDS)

Field-emission scanning electron microscopy (FE-SEM, Hitachi Regulus 8100), Hitachi High-Tech Corporation, Tokyo, Japan, was employed to characterize the surface and cross-sectional morphologies of the samples. Energy-dispersive X-ray spectroscopy (EDS), Hitachi High-Tech Corporation, Tokyo, Japan, attached to the FE-SEM was used to analyze the elemental composition and distribution. The pore sizes and layer thicknesses were obtained via statistical analysis of the SEM images using ImageJ software (National Institutes of Health, Bethesda, MD, USA; Laboratory for Optical and Computational Instrumentation, University of Wisconsin-Madison, Madison, WI, USA).

#### 2.3.3. Transmission Electron Microscopy (TEM and HRTEM)

The detailed microstructure and lattice information of the ZIS/SIS heterojunction were investigated by transmission electron microscopy (TEM) and high-resolution transmission electron microscopy (HRTEM) on an FEI Talos F200X G2 instrument, Thermo Fisher Scientific, Waltham, MA, USA.

#### 2.3.4. X-Ray Photoelectron Spectroscopy (XPS)

X-ray photoelectron spectroscopy (XPS, Thermo Scientific K-Alpha), Thermo Fisher Scientific, Waltham, MA, USA, was used to determine the surface chemical states and elemental compositions of the samples. All binding energies were calibrated using the C 1s peak at 284.8 eV as the internal standard.

#### 2.3.5. UV-Visible Diffuse Reflectance Spectroscopy (UV-Vis DRS)

UV-visible diffuse reflectance spectroscopy (UV-vis DRS) was performed on a TU-1901 spectrophotometer equipped with an integrating sphere, Purkinje General Instrument Co., Ltd., Beijing, China. The test wavelength range was 200–800 nm for analyzing the optical absorption properties.

#### 2.3.6. Electrochemical Characterization

**Figure 2:**
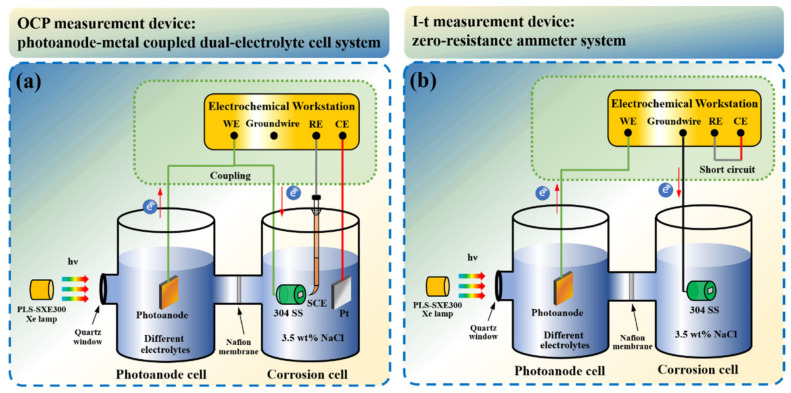
(**a**) Schematic diagram of a device for measuring open circuit potential. (**b**) Schematic illustration of testing device for measuring the photoinduced current density.

PECCP measurements were conducted on a CHI 760E electrochemical workstation, Shanghai Chenhua Instrument Co., Ltd., Shanghai, China, as shown in Figure 2. The Open Circuit Potential (OCP) testing setup consisted of three main components: the electrochemical workstation, a dual-compartment cell (photoanode cell and corrosion cell), and an Xe lamp light source (PLS-SXE300, Beijing Perfectlight Technology Co., Ltd., Beijing, China). The experiments employed a dual-compartment cell separated by a Nafion membrane, with 304 SS as the coupled electrode, to evaluate the photoanode performance in three different electrolytes: 3.5 wt% NaCl, 0.1 M Na_2_S_2_O_3_ and Na_2_S/NaOH solutions. The Nafion membrane prevents direct diffusion of sacrificial agents while maintaining ionic conductivity, ensuring the cathodic protection originates solely from photogenerated electrons rather than chemical reactions. The three-electrode system was configured as follows: the working electrode (WE) was formed by connecting the photoanode (in various electrolytes) to the protected metal (in 3.5 wt% NaCl) via a copper wire; a saturated calomel electrode (SCE) with a Luggin capillary (was filled with saturated KCl solution) and a Pt electrode served as the reference electrode (RE) and counter electrode (CE), respectively. OCP tests were performed under intermittent visible light illumination with 200 s light on/off cycles. A 420 nm cutoff filter was installed on the 300 W Xe lamp to simulate visible light irradiation (λ ≥ 420 nm), and the light intensity was adjusted to 100 mW·cm^−2^ using a calibrated silicon photodiode. A quartz window ensured high light transmission to the photoanode surface. I-t curves were measured at zero bias with 50 s light on/off intervals. For I-t measurements, the WE was connected to the photoanode and the ground wire to the protected metal, with RE and CE short-circuited, enabling the workstation to function as a zero-resistance ammeter for measuring unpolarized current density without polarization effects. Both Electrochemical Impedance Spectroscopy (EIS), Mott-Schottky plot (M-S) and Linear Sweep Voltammetry (i-V) measurements were carried out in 0.1 M Na_2_SO_4_ electrolyte using a standard three-electrode configuration. Before each EIS measurement, the working electrode was kept at open circuit potential (OCP) in the dark for 30 min to reach a stable state. The EIS tests covered a frequency range from 0.01 to 100,000 Hz with an AC amplitude of 5 mV. The M-S measurements were conducted in dark conditions at frequencies of 1000, 1500, and 2000 Hz with 10 mV AC amplitude, scanning from −1.5 V to 0.4 V. The i–V curves were recorded with a scan rate of 0.02 V/s over a potential range from –1.5 V to 0 V (vs. SCE). All measurements used a saturated calomel electrode (SCE) as the reference electrode and a platinum plate as the counter electrode.

## 3. Results

### 3.1. Structure, Morphology and Surface State Analyses

**Figure 3:**
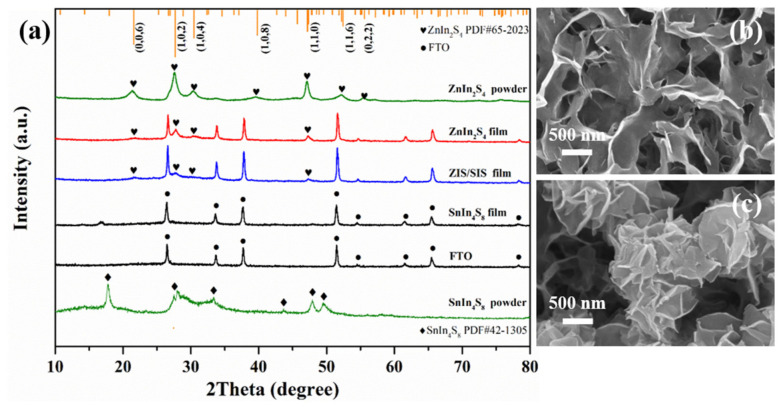
(**a**) XRD patterns of the bare FTO substrate, SnIn_4_S_8_ film, SnIn_4_S_8_ powder, ZnIn_2_S_4_ film, ZIS/SIS film, and ZnIn_2_S_4_ powder. (**b**) SEM image of the SnIn_4_S_8_ film. (**c**) SEM image of the SnIn_4_S_8_ powder.

The crystal structures of SnIn_4_S_8_ powder, SnIn_4_S_8_ thin film, ZIS/SIS heterojunction thin film, ZnIn_2_S_4_ thin film, and ZnIn_2_S_4_ powder were systematically characterized by XRD. As shown in Figure 3a, a direct visual comparison between the XRD pattern of the bare FTO substrate and that of the SnIn_4_S_8_ thin film reveals that the two curves are nearly identical, with no distinct crystalline diffraction peaks observable for the SnIn_4_S_8_ thin film. This phenomenon may be attributed to two main factors: the low crystallinity of the thin film under the given synthesis conditions, and the possible preferential orientation growth along certain crystal planes induced by the FTO substrate, which weakened the diffraction signals from other planes. The low crystallinity of the SnIn_4_S_8_ thin film is further supported by the corresponding SEM image (Figure 3b), which shows a porous nanoflake array architecture. In contrast, the SnIn_4_S_8_ powder sample, prepared under identical synthesis conditions, exhibits a well-defined microsphere morphology with an average diameter of approximately 1.6 μm (Figure 3c). This pronounced morphological difference correlates well with their distinct crystallographic behaviors observed by XRD. The SnIn_4_S_8_ powder sample showed clear diffraction peaks that matched well with the cubic phase SnIn_4_S_8_ standard PDF card (PDF#42-1305) [43]. Five major diffraction peaks were observed at 18.1°, 27.6°, 28.2°, 47.9°, and 49.7°, which can be assigned to the (202), (311), (400), (440), and (531) crystal planes of cubic SnIn_4_S_8_, collectively confirming the successful synthesis of the cubic SnIn_4_S_8_ material. The ZnIn_2_S_4_ powder sample exhibited distinct diffraction peaks at 2θ values of 21.6°, 27.7°, 30.4°, 39.8°, 47.2°, 52.4°, and 55.6°. Comparison with the standard card (JCPDS No. 65-–2023) confirmed that these peaks correspond to the (006), (102), (104), (108), (110), (116), and (022) planes of hexagonal ZnIn_2_S_4_, demonstrating the well-defined hexagonal crystal structure of the as-synthesized ZnIn_2_S_4_ powder [42]. Notably, the diffraction peak at 27.7°, corresponding to the (102) plane, showed the highest intensity, suggesting a possible preferred crystal orientation. In the thin film samples, both the pure ZnIn_2_S_4_ film and the ZIS/SIS heterojunction film deposited on FTO substrates exhibited characteristic diffraction peaks of ZnIn_2_S_4_. It is noteworthy that no characteristic peaks of SnIn_4_S_8_ were detected in the XRD pattern of the ZIS/SIS heterojunction film aside from those from the FTO substrate and ZnIn_2_S_4_. This absence is consistent with the low crystallinity of the pure SnIn_4_S_8_ thin film, as depicted in Figure 3a. However, the successful synthesis of SnIn_4_S_8_ had been confirmed by its powder XRD results as mentioned above. Further evidence for the successful construction of the ZIS/SIS heterojunction was provided by subsequent characterization, including EDS and XPS, which revealed the elemental composition and chemical states.

**Figure 4:**
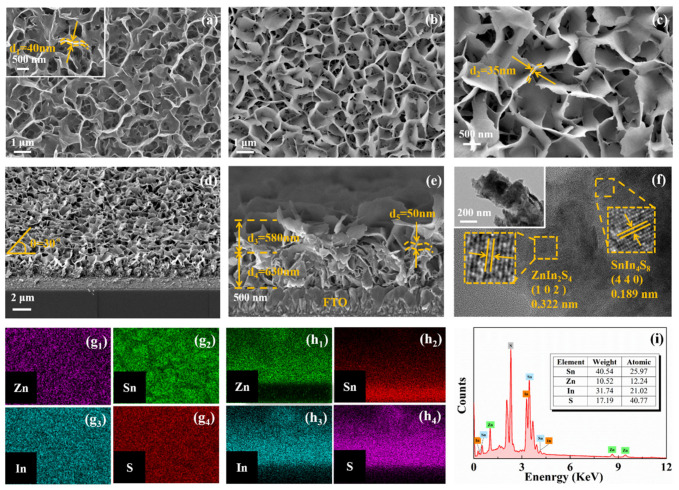
Microstructure and elemental distribution characterization of SnIn_4_S_8_, ZnIn_2_S_4_, and ZIS/SIS heterojunction. (**a**) Top-view SEM image of SnIn_4_S_8_, thin film, (**b**) top-view SEM image of ZnIn_2_S_4_ thin film, (**c**) top-view SEM image of ZIS/SIS heterojunction, (**d**) 30° tilted-view SEM image of ZIS/SIS heterojunction, (**e**) cross-sectional SEM image of ZIS/SIS heterojunction, (**f**) TEM and HRTEM images of ZIS/SIS heterojunction, (**g_1_**) EDS elemental mapping of Zn corresponding to panel (**c**), (**g_2_**) EDS elemental mapping of Sn corresponding to panel (**c**), (**g_3_**) EDS elemental mapping of In corresponding to panel (**c**), (**g_4_**) EDS elemental mapping of S corresponding to panel (**c**), (**h_1_**) EDS elemental mapping of Zn corresponding to panel (**e**), (**h_2_**) EDS elemental mapping of Sn corresponding to panel (**e**), (**h_3_**) EDS elemental mapping of In corresponding to panel (**e**), (**h_4_**) EDS elemental mapping of S corresponding to panel (**e**), (**i**) EDS spectra corresponding to (**c**).

SEM characterization revealed the porous structural features and elemental distribution of the ZIS/SIS heterojunction. As shown in Figure 4a, the SnIn_4_S_8_ film displays a nanoplate-assembled porous foam architecture with an average thickness of 40 nm, whose open-channel structure provides an ideal template for ZnIn_2_S_4_ heteroepitaxy. Figure 4b demonstrates that the ZnIn_2_S_4_ component possesses a porous nanoflower morphology with serrated edges, significantly enhancing light harvesting efficiency. The heterojunction construction is illustrated in Figure 4c, featuring a continuous SnIn_4_S_8_ porous network as the bottom layer and a uniformly grown ZnIn_2_S_4_ nanoplates array on top. The 30° tilted view (Figure 4d) clearly shows their interlocked interface structure, facilitating charge transfer. The unique 3D interlocked architecture is not merely a morphological curiosity but a cornerstone of its design for high-performance and sustainable PECCP. This interpenetrating network creates continuous charge transfer highways, drastically shortening the diffusion path for photogenerated electrons and providing a large, robust interface for charge separation—a critical feature for maintaining long-term protection stability in fluctuating marine environments. Cross-sectional analysis (Figure 4e) reveals a layered architecture: a 630 ± 25 nm SnIn_4_S_8_ layer and a 580 ± 20 nm ZnIn_2_S_4_ layer sequentially deposited on FTO substrate, with an interfacial transition zone of ~50 nm indicating possible interdiffusion. Elemental mapping confirmed successful heterojunction formation. The microstructure of the ZIS/SIS heterojunction was further investigated by TEM and HRTEM. The TEM image in Figure 4f clearly displays an interlocked and cross-linked structure formed between ZnIn_2_S_4_ and SnIn_4_S_8_. HRTEM results further reveal the lattice characteristics of the composite, where measured spacings of 0.322 nm and 0.189 nm correspond to the (102) plane of ZnIn_2_S_4_ and the (440) plane of SnIn_4_S_8_, respectively.

In addition, elemental mapping was performed to examine the spatial distribution of constituent elements. Top-view EDS mapping (Figure 4(g_1_–g_4_)) shows a homogeneous nanoscale distribution of Zn, Sn, In, and S elements. Cross-sectional mapping (Figure 4(h_1_–h_4_)) reveals spatially resolved elemental distributions corresponding to respective components, with interfacial gradient variations corroborating SEM observations. This moderate elemental interdiffusion during heterojunction formation may facilitate charge carrier separation. The EDS spectrum of the ZIS/SIS heterojunction is presented in Figure 4i. The atomic percentages of Sn, Zn, In, and S elements are approximately 25.97%, 12.24%, 21.02%, and 40.77%, respectively. The Sn element originates primarily from SnIn_4_S_8_, while Zn is derived from ZnIn_2_S_4_. Both In and S are common constituents in the two components. The presence of these elements in the measured ratios strongly confirms the successful formation of the ZIS/SIS heterojunction.

**Figure 5:**
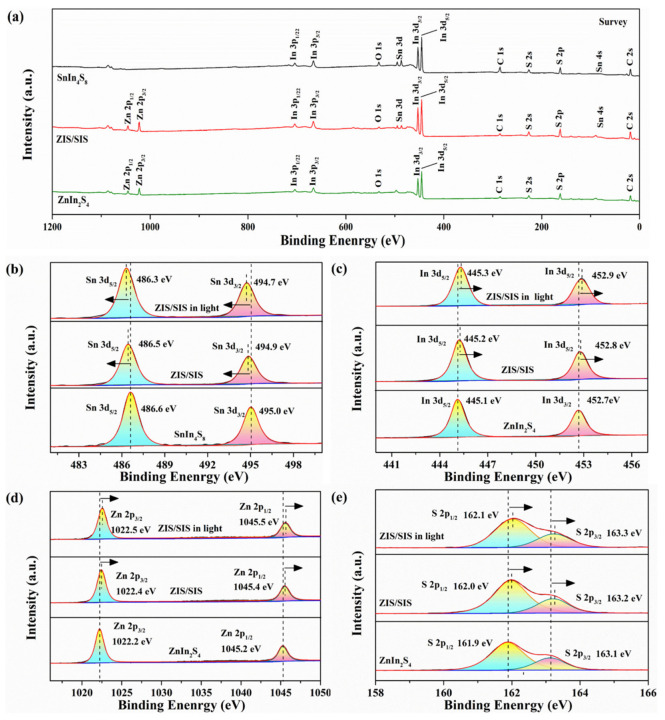
(**a**) Full scan survey XPS spectra and high-resolution ISI-XPS spectra of (**b**) Sn 3d, (**c**) In 3d, (**d**) Zd 3d and (**e**) S 2p of pristine SnIn_4_S_8_, pristine ZnIn_2_S_4_ and ZIS/SIS.

The chemical composition and interfacial charge transfer behavior of the ZIS/SIS heterojunction were systematically investigated by XPS. To ensure measurement accuracy, all XPS spectra were calibrated using the C 1 s peak (284.8 eV) from adventitious carbon contamination as an internal reference. Figure 5a displays the XPS survey spectrum, where characteristic peaks corresponding to Sn 3d, In 3d, Zn 2p, and S 2p were clearly observed in the ZIS/SIS heterojunction. Combined with EDS elemental mapping results, this confirms the coexistence of both SnIn_4_S_8_ and ZnIn_2_S_4_ phases. The binding energy shifts in XPS spectra directly reflect variations in electron density, where a decrease in electron density leads to an increase in binding energy [44]. This principle enables the detection of charge carrier transfer directions in heterojunction photocatalysts. The high-resolution Sn 3d XPS spectrum in Figure 5b demonstrates that the Sn 3d peaks in the ZIS/SIS heterojunction exhibit a 0.1 eV negative shift (486.5 eV for Sn 3d_5/2_ and 494.9 eV for Sn 3d_3/2_) compared to pristine SnIn_4_S_8_ (486.6 eV and 495.0 eV, respectively), suggesting an enhanced electron density around Sn^4+^ species. Figure 5c presents the In 3d fine spectrum, showing a 0.1 eV positive shift for both In 3d_5/2_ (445.2 eV) and In 3d_3/2_ (452.8 eV) in ZIS/SIS relative to pure ZnIn_2_S_4_ (445.1 eV and 452.7 eV), suggesting decreased electron density of In^3+^. The Zn 2p high-resolution spectrum in Figure 5d further demonstrates a 0.2 eV increase in binding energy for both Zn 2p_3/2_ (1022.4 eV) and Zn 2p_1/2_ (1045.4 eV) compared to pure ZnIn_2_S_4_ (1022.2 eV and 1045.2 eV), confirming reduced electron cloud density around Zn^2+^ ions. Similarly, as shown in Figure 5e, the S 2p spectrum exhibits a 0.1 eV positive shift for both S 2p_1/2_ (162.0 eV) and S 2p_3/2_ (163.2 eV) peaks relative to the pristine phases (161.9 eV and 163.1 eV), further supporting this electron transfer trend. These binding energy variations demonstrate that under Fermi level equilibration, the electron density decreases for Zn, In, and S elements in ZnIn_2_S_4_ (electron donor) while increasing for Sn in SnIn_4_S_8_ (electron acceptor). The electron migration between the two semiconductors also leads to changes in the binding energy of specific elements [45,46], confirming the transfer of photogenerated electrons from ZnIn_2_S_4_ to SnIn_4_S_8_ and the formation of a space charge layer at the interface ZnIn_2_S_4_ to SnIn_4_S_8_ [19]. 

Furthermore, in situ irradiated XPS (ISI-XPS) measurements provide direct evidence for light-induced charge separation. Under photoexcitation, the ZIS/SIS heterojunction exhibits additional positive shifts in Zn 2p, In 3d, and S 2p binding energies compared to the dark condition, while the Sn 3d peaks show further negative shifts. This confirms the migration of photogenerated electrons from ZnIn_2_S_4_ to SnIn_4_S_8_. The overall XPS results thereby compellingly demonstrate that the interfacial charge transfer in the ZIS/SIS heterojunction follows a type-II mechanism, which effectively suppresses electron-hole recombination and significantly enhances charge separation efficiency.

### 3.2. Optical Absorption and Energy Band Structures

**Figure 6:**
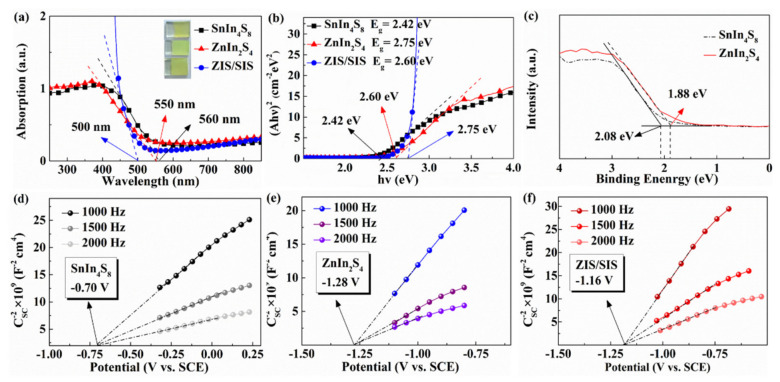
(**a**) UV-vis diffuse reflectance spectra, (**b**) Tauc plots, (**c**) XPS valence band spectra, M-S curves of (**d**) SnIn_4_S_8_, (**e**) ZnIn_2_S_4_, (**f**) ZIS/SIS under dark conditions.

Figure 6a presents the UV-vis absorption spectra of the as-prepared samples. The absorption edge of the ZIS/SIS heterojunction is located between those of SnIn_4_S_8_ (560 nm) and ZnIn_2_S_4_ (500 nm). Compared with pristine SnIn_4_S_8_, the ZIS/SIS heterojunction exhibits a blue-shifted absorption edge, indicating a reduced visible-light response capability, which may be attributed to the band gap widening induced by the incorporation of ZnIn_2_S_4_. Nevertheless, the ZIS/SIS heterojunction still maintains a broad absorption range in the visible region, suggesting its potential for photoelectrochemical conversion. As shown in Figure 6b, the band gaps (E_g_) of SnIn_4_S_8_, ZnIn_2_S_4_, and ZIS/SIS are determined to be 2.42 eV, 2.75 eV, and 2.60 eV, respectively, through Tauc plot analysis based on the Kubelka-Munk transformation:

(1) $${\left(\alpha hv\right)}^{n}=A\left(hv-E_{g}\right)$$

where *α* is the absorption coefficient, hv is the photon energy, *A* is a constant, and the exponent *n* = 2 was used for the direct band gap transitions of both SnIn_4_S_8_ and ZnIn_2_S_4_ semiconductors.

The electronic band alignment of the semiconductors was systematically investigated. Based on the XPS valence band spectra presented in Figure 6c, the valence band maximum (VBM) positions of SnIn_4_S_8_ and ZnIn_2_S_4_ were determined to be 2.08 eV and 1.88 eV, respectively, relative to the Fermi level. The corresponding valence band (VB) edge potentials versus the normal hydrogen electrode (NHE) were calculated using the following relationship [47]:

(2) $$E_{VB,\;NHE}\;=\;\Phi_{spectrometer}\;+\;E_{VB,\;XPS}\;-\;4.44$$

where *E_VB, NHE_* is the VB edge potential relative to NHE (eV), *Φ_spectrometer_* is the spectrometer work function (4.2 eV), and *E_VB, XPS_* is the VB binding energy obtained from XPS measurements. This yields *E_VB_* values of +1.84 V vs. NHE for SnIn_4_S_8_ and +1.64 V vs. NHE for ZnIn_2_S_4_.

The E_g_ values were derived from the Tauc plots in Figure 6b, giving 2.42 eV for SnIn_4_S_8_ and 2.75 eV for ZnIn_2_S_4_. The conduction band (CB) minimum potentials were then estimated using the equation [47]:

(3) $$E_{CB}\;=\;E_{VB,\;NHE}\;-\;E_{g}$$

where *E_CB_* represents the conduction band edge potential relative to NHE. Accordingly, the calculated *E_CB_* for SnIn_4_S_8_ is −0.58 V vs. NHE, while that for ZnIn_2_S_4_ is −1.11 V vs. NHE.

To further probe the semiconductor-electrolyte interface properties and charge transfer dynamics, Mott-Schottky (M-S) analysis was performed (Figure 6d–f). To ensure accuracy and account for frequency dispersion, measurements were conducted at three discrete frequencies (1000, 1500, and 2000 Hz), and the final E_fb_ value for each sample was taken from the convergent point of these three extrapolated lines. Linear extrapolation of the M-S plots yields flat-band potentials (E_fb_) of −0.70 V for SnIn_4_S_8_, −1.28 V for ZnIn_2_S_4_, and −1.16 V (vs. SCE) for ZIS/SIS. The results demonstrated that all specimens exhibited positive slopes in the M-S plots, confirming their n-type semiconductor characteristics [48]. Notably, the ZIS/SIS heterojunction shows a substantial negative shift (460 mV) in flat-band potential compared to pristine SnIn_4_S_8_, indicating the formation of efficient charge transfer channels at the heterointerface. The more negative E_fb_ of ZnIn_2_S_4_, consistent with its wider band gap (E_g_ ≈ 2.75 eV) and higher density of defect states, positions it favorably for electron donation within the heterojunction. This band alignment, previously confirmed by the ISI-XPS results (Figure 5), creates a type-II heterostructure that produces two crucial effects [49]: firstly, it generates a strong built-in electric field in the space charge region, providing an additional driving force for the separation of photogenerated carriers; secondly, it optimizes the electron transfer pathway, as evidenced by the M-S potential shift and in situ XPS data, enabling photogenerated electrons to rapidly migrate to the electrode surface and participate in reactions.

**Figure 7:**
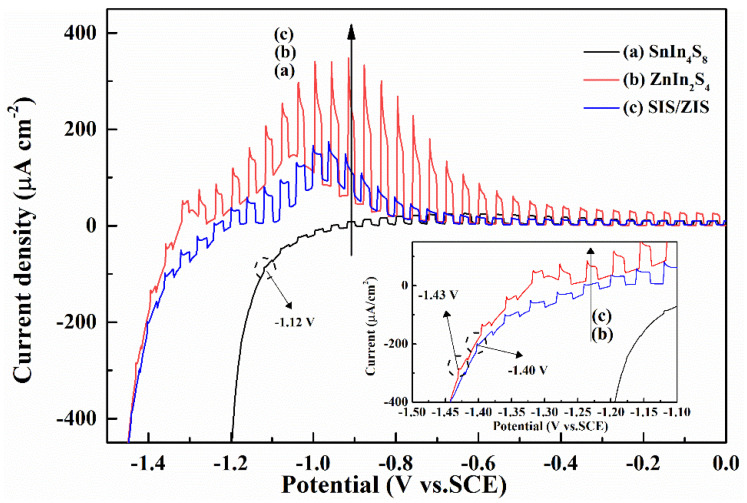
i-V plots under intermittent light of SnIn_4_S_8_, ZnIn_2_S_4_, and the ZIS/SIS heterojunction.

The photoinduced i-V curves of SnIn_4_S_8_, ZnIn_2_S_4_, and the ZIS/SIS heterojunction were measured under intermittent illumination. As shown in Figure 7, the ZIS/SIS heterojunction exhibits a significantly enhanced photocurrent compared to SnIn_4_S_8_ and ZnIn_2_S_4_. The net photocurrent density (ΔI), defined as the difference between the current density under illumination and in the dark at a given potential, was used to quantitatively evaluate the performance. At a bias voltage of −0.92 V (vs. SCE), the ZIS/SIS heterojunction achieved a ΔI of approximately 285.4 μA·cm^−2^, which is about 23.0 times that of SnIn_4_S_8_ (≈12.4 μA·cm^−2^) and 3.6 times that of ZnIn_2_S_4_ (≈78.5 μA·cm^−2^). This remarkable enhancement clearly demonstrates the facilitated separation efficiency of photogenerated charges in the heterojunction structure. The potential at which the photocurrent transitions is closely related to the Fermi level (E_F_) of the semiconductor and is referred to as the threshold bias potential [50]. As indicated in Figure 7, this potential is approximately −1.12 V for pristine SnIn_4_S_8_ and −1.40 V for ZnIn_2_S_4_, while it shifts negatively to −1.43 V for the ZIS/SIS heterojunction. This negative shift suggests that the incorporation of ZnIn_2_S_4_ modifies the electronic structure, causing the E_F_ of SnIn_4_S_8_ to move toward a more negative potential. Such an adjustment in the energy band structure not only enhances the reducing capability of photogenerated electrons but also further promotes the separation of photogenerated charge carriers. In summary, as illustrated in Figure 7, the constructed ZIS/SIS heterojunction exhibits significantly improved photoelectrochemical conversion performance, which can be attributed to its superior electron-hole separation efficiency and accelerated interfacial carrier transfer.

**Figure 8:**
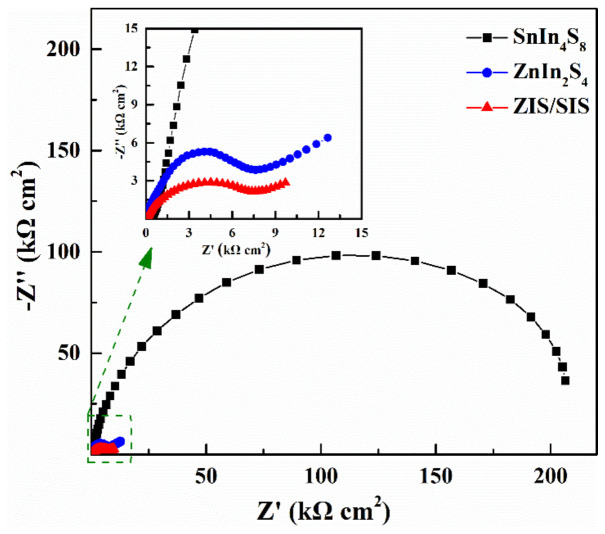
EIS spectra of pristine SnIn_4_S_8_, ZnIn_2_S_4_, and ZIS/SIS photoelectrodes in 0.1 M Na_2_SO_4_ solution under visible light illumination.

EIS analysis under simulated visible light irradiation (Figure 8) was systematically conducted to investigate the charge transfer and recombination processes in SnIn_4_S_8_, ZnIn_2_S_4_, and ZIS/SIS heterojunction electrodes. Nyquist plots showed clear differences in electrochemical behavior among the samples. SnIn_4_S_8_ demonstrated a single semicircular arc characteristic of a charge-transfer-controlled process. In contrast, both ZnIn_2_S_4_ and ZIS/SIS exhibited more complex impedance patterns, consisting of a high-frequency semicircle combined with a low-frequency Warburg tail. This distinct impedance behavior indicated that the heterostructure fundamentally altered the charge transport mechanism, transitioning from purely interfacial charge-transfer control to a hybrid process involving both interfacial transfer and bulk diffusion. According to electrochemical kinetics theory, the semicircle radius in the high-frequency region directly correlates with the charge transfer resistance (R_ct_) at the electrode-electrolyte interface, where a smaller radius reflects more efficient charge transfer [51]. The impedance arc radii followed the order: SnIn_4_S_8_ > ZnIn_2_S_4_ > ZIS/SIS, demonstrating that the heterojunction interface established highly efficient charge transport pathways, substantially reducing the interfacial charge transfer barrier. This facilitated rapid photogenerated electron migration across the heterointerface from ZnIn_2_S_4_ to SnIn_4_S_8_. These results confirm that the ZIS/SIS heterostructure enhances charge separation and transport efficiency through optimized interfacial band alignment and built-in electric field formation. The synergistic effect between energy level matching and interfacial engineering promotes superior charge carrier dynamics in the heterojunction system.

### 3.3. Photocathodic Protection Performance for 304 SS

In the field of PECCP, both photogenerated OCP and photocurrent density serve as crucial parameters for evaluating material performance. The photoelectrochemical behaviors of SnIn_4_S_8_, ZnIn_2_S_4_ and their heterojunction coupled with 304 SS were systematically investigated in three different electrolyte systems containing hole scavengers (3.5 wt% NaCl, 0.1 M Na_2_S_2_O_3_, and 0.1 M Na_2_S + 0.2 M NaOH), which provides fundamental understanding of their cathodic protection mechanisms and performance differences. This multi-electrolyte approach is essential for a comprehensive understanding. The use of highly reductive scavengers (Na_2_S/NaOH) allows us to probe the maximum charge separation capability of the heterojunction itself by rapidly consuming holes. Conversely, testing in neutral NaCl and natural seawater, devoid of such scavengers, provides a true and critical assessment of the material’s practical viability for marine applications, where such additives are absent. The superior performance of ZIS/SIS across this spectrum of conditions unequivocally demonstrates both its intrinsic excellence and its robustness in realistic environments. From a corrosion thermodynamics perspective, the OCP value represents a critical criterion for assessing metal corrosion tendency. More negative potential shift indicates lower thermodynamic driving force for corrosion and better anti-corrosion performance. From corrosion kinetics standpoint, the photocurrent density reflects the separation and transport efficiency of photogenerated carriers. Higher photocurrent density corresponds to superior photoelectric conversion efficiency and stronger cathodic protection current for metal substrates [52]. The combination of these two parameters enables comprehensive evaluation of both thermodynamic protection capability and kinetic protection efficiency of photoelectrochemical cathodic protection materials.

**Figure 9:**
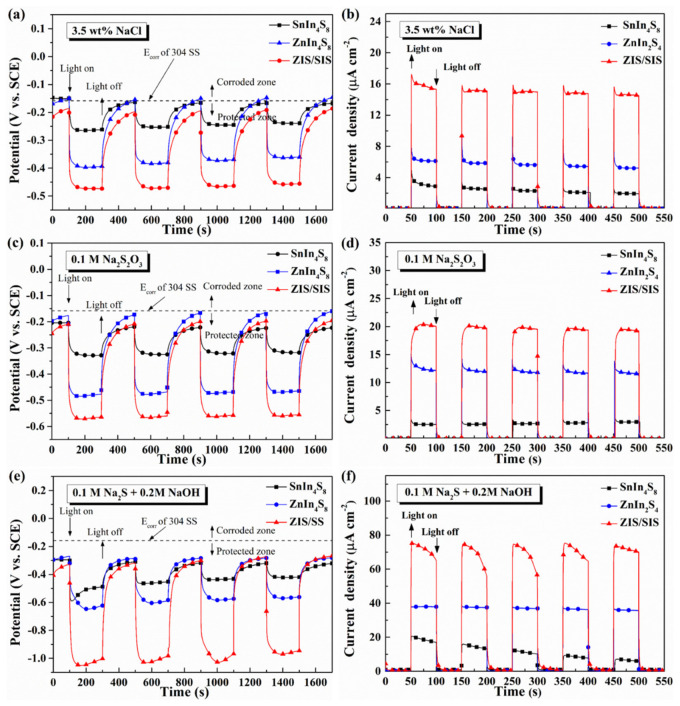
Photoelectrochemical cathodic protection performance of the SnIn_4_S_8_, ZnIn_2_S_4_ and ZIS/SIS photoanodes coupled with 304 SS electrode in different electrolyte systems: (**a**,**b**) OCP variation and photocurrent density response in 3.5 wt% NaCl solution; (**c**,**d**) in 0.1 M Na_2_S_2_O_3_ solution; (**e**,**f**) in 0.1 M Na_2_S + 0.2 M NaOH solution.

Under intermittent light illumination, Figure 9a,b present the OCP variation and photocurrent-time (I-t) curves of SnIn_4_S_8_, ZnIn_2_S_4_ and ZIS/SIS photoanodes coupled with 304 SS electrode in 3.5 wt% NaCl solution. As shown in Figure 9a, the OCPs of single-component SnIn_4_S_8_ and ZnIn_2_S_4_ photoanodes stabilized at −0.25 V and −0.37 V respectively under illumination in simulated marine environment (3.5 wt% NaCl), while the ZIS/SIS heterojunction exhibited more pronounced negative potential shift, stabilizing at −0.47 V. This value is significantly lower than the corrosion potential of 304 stainless steel (−0.18 V), confirming the enhanced cathodic protection capability of the heterostructure. Figure 9b further reveals the charge separation efficiency through photocurrent density measurements. The single-component SnIn_4_S_8_ and ZnIn_2_S_4_ showed limited photocurrent densities of merely 2.33 μA∙cm^−2^ and 5.60 μA∙cm^−2^, respectively, both displaying gradual decay trends. In contrast, the heterojunction material maintained a stable photocurrent density at 15.22 μA∙cm^−2^, representing 6.5-fold and 2.7-fold enhancements compared to SnIn_4_S_8_ and ZnIn_2_S_4,_ respectively. This significant improvement originates from the type-II band alignment at the heterojunction interface, which effectively promotes spatial separation of photogenerated carriers while the optimized interfacial charge transfer pathway substantially reduces electron-hole recombination probability. The outstanding performance of the ZIS/SIS heterojunction in the 3.5 wt% NaCl solution, which simulates a harsh marine environment, is of paramount importance for practical applications. The significant negative shift in the OCP to −0.47 V and the stable photocurrent density of 15.22 μA·cm^−2^ directly demonstrate its capability to provide effective cathodic protection and enhance the durability of 304 SS against marine corrosion. This performance is exceptional in the context of practical marine applications, as it is achieved in a neutral chloride environment without relying on sacrificial agents, thereby simulating real-world protection scenarios.

Figure 9c demonstrates the distinct OCP performance differences among various photoanodes in 0.1 M Na_2_S_2_O_3_ electrolyte. SnIn_4_S_8_ exhibited a potential drop of 120 mV but showed obvious potential recovery, indicating limited charge separation efficiency. ZnIn_2_S_4_ displayed superior performance with a 290 mV negative potential shift. The ZIS/SIS heterojunction achieved the maximum potential drop of 380 mV, stabilizing at −0.57 V. The photocurrent response characteristics in Figure 9d reveal that SnIn_4_S_8_ presented typical “spike-decay” behavior with a steady-state current of only 2.6 μA∙cm^−2^, while ZnIn_2_S_4_ showed an improved steady-state current of 11.96 μA∙cm^−2^. Remarkably, the heterojunction material demonstrated unique “spike-free steady-maintaining” characteristics with stable current output at 19.76 μA∙cm^−2^. This exceptional performance stems from the selective enrichment of sulfides at the heterojunction interface, which strengthens the interfacial electric field through a thermodynamically driven adsorption process, enabling immediate carrier separation and rapid transport while avoiding the common carrier accumulation-release hysteresis in conventional materials.

As illustrated in Figure 9e,f, the performance differences became more pronounced in a strongly reductive 0.1 M Na_2_S + 0.2 M NaOH electrolyte. The stability was quantitatively evaluated over four on-off cycles. SnIn_4_S_8_ suffered from severe photodegradation, with its OCP shift decaying by 64.1% (from 290 mV to 104 mV) and its photocurrent decaying by 71.0% (from 20.62 μA·cm^−2^ to 5.98 μA·cm^−2^). This rapid degradation resulted from two key factors: severe surface corrosion under strong alkaline conditions coupled with insufficient hole consumption, leading to rapid photocorrosion; and the aggravated surface instability and cumulative defect formation under continuous illumination. In contrast, the degradation was much milder in a neutral NaCl solution due to the absence of such corrosive environment. ZnIn_2_S_4_ exhibited better stability, retaining 78.9% of its initial OCP shift (from 380 mV to 300 mV) and 89.8% of its initial photocurrent (from 39.13 μA·cm^−2^ to 35.13 μA·cm^−2^). Most notably, the ZIS/SIS heterojunction demonstrated the most outstanding performance and stability. It achieved a remarkable initial OCP shift of 720 mV and retained 96.7% of this value (696 mV) after four cycles, reaching a final potential of −1.05 V. Similarly, its photocurrent density showed excellent stability, maintaining 73.61 μA·cm^−2^ (97.7% retention of the initial 75.35 μA·cm^−2^) after four cycles. More importantly, the construction of ZIS/SIS heterojunction dramatically suppressed the severe degradation observed in pure SnIn_4_S_8_. This performance enhancement can be attributed to multiple synergistic effects: the heterojunction structure promotes directional carrier migration, thus effectively separating electron-hole pairs and minimizing photocorrosion; S^2−^ from Na_2_S effectively consumes photogenerated holes; and the alkaline-induced surface hydroxylation optimizes charge transport pathways. All these factors collectively achieve highly efficient and stable photoelectrochemical protection.

**Figure 10:**
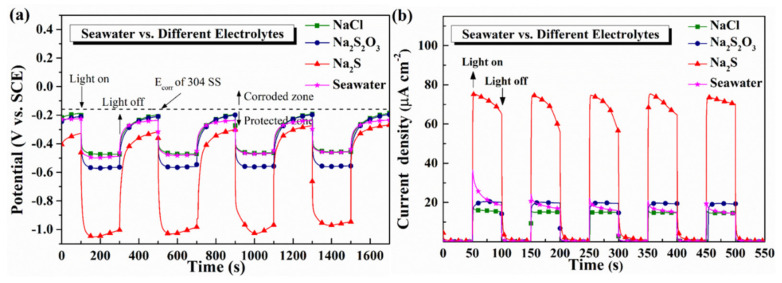
Comparison of the (**a**) OCP and (**b**) photocurrent density response for the ZIS/SIS heterojunction coupled with 304 SS in natural seawater versus three other electrolyte systems.

Comparative analysis across multiple electrolyte systems confirms the consistently superior performance of ZIS/SIS heterojunction over single-component materials (see Figure 10). Regarding OCP, the Na_2_S/NaOH system showed optimal performance with 720 mV negative shift, being 2.5-fold and 1.9-fold greater than those in NaCl and Na_2_S_2_O_3_ systems, respectively. Notably, in natural seawater, the OCP instantly shifted by 275 mV upon illumination, reaching −0.49 V, which is more negative than that in NaCl solution. Although the potential gradually shifted positively over successive light cycles, the overall OCP curve remained close to that observed in NaCl. Similarly, the I–t response in seawater showed an initial photocurrent density of 35.11 μA∙cm^−2^ under illumination, higher than that in NaCl, but it gradually decreased with cycling. By the final cycle, the I–t curve nearly coincided with that in NaCl, indicating that the heterojunction retains considerable activity even in the complex ionic environment of real seawater, though its performance is influenced by the absence of sacrificial agents. The photocurrent density across all systems followed the trend of Na_2_S/NaOH > Na_2_S_2_O_3_ > NaCl ≈ Natural Seawater. The Na_2_S/NaOH system achieved a maximum of 72.27 μA∙cm^−2^, which is 4.7 times greater than that in the neutral NaCl solution. The exceptional performance of the ZIS/SIS heterojunction in the scavenger-free 3.5 wt% NaCl solution is of paramount practical significance for sustainable marine corrosion control. The significant negative shift in the OCP to −0.47 V and the stable photocurrent density of 15.22 μA·cm^−2^ are achieved without relying on any sacrificial agents, directly demonstrating its capability for direct solar energy conversion into effective and sustainable cathodic protection for 304 SS in simulated marine environments. The slight potential positive shift observed in the strongly reductive system may be related to transient concentration polarization caused by high-concentration carrier transport, while the overall cycling stability remained satisfactory. Various active components in different electrolytes (e.g., S_2_O_3_^2−^, S^2−^) further improved interfacial reaction efficiency through surface modification and hole scavenging pathways. Particularly in the strongly reductive system, multiple synergistic effects significantly enhanced both photoelectric conversion efficiency and stability of the heterojunction material.

The superior and robust performance of the ZIS/SIS heterojunction across this spectrum of conditions, and particularly in scavenger-free environments, unequivocally demonstrates its dual advantage: exceptional intrinsic charge separation properties and practical viability for sustainable corrosion control in real marine settings. Notably, the stable performance in the scavenger-free 3.5 wt% NaCl solution holds exceptional practical relevance for sustainable technology. The achieved OCP shift and photocurrent density are fully sufficient to provide effective cathodic protection, proving the material’s capability to function autonomously under conditions that directly simulate a real marine environment, thereby fulfilling the core requirement of sustainability by eliminating the need for chemical additives.

**Figure 11:**
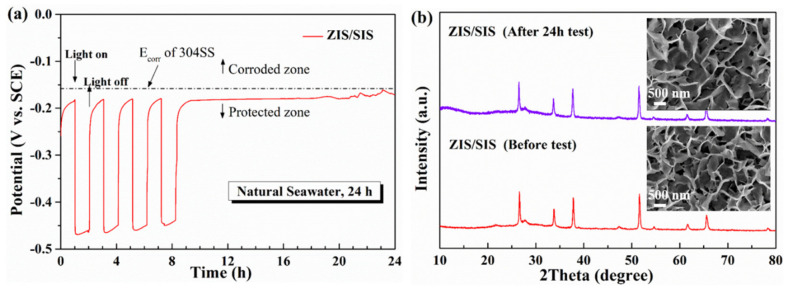
(**a**) Long-term OCP evolution of the ZIS/SIS heterojunction coupled with 304 SS in natural seawater, and (**b**) XRD patterns and SEM images before and after the 24 h OCP test.

The stability of the heterojunction is critical for long-term photogenerated cathodic protection. To evaluate the long-term performance and stability of the ZIS/SIS heterojunction in natural seawater, a 24 h OCP test was conducted. As shown in Figure 11a, upon illumination, the OCP immediately dropped to −0.47 V, with a potential drop of 285 mV. After 8 h of intermittent light cycling (1 h light/dark intervals, totaling 4 cycles), the potential drop showed a slight decrease but remained largely stable and well below the corrosion potential of 304 SS. Notably, even after the light was turned off, the system continued to provide effective protection to 304 SS for another 16 h in the dark, demonstrating its remarkable stability. To further investigate the structural stability of the ZIS/SIS heterojunction after testing, XRD and SEM analyses were performed. The XRD pattern (Figure 11b) revealed no significant emergence of new peaks, indicating that the crystalline structure of ZIS/SIS remained intact under prolonged illumination. In addition, SEM images showed no obvious morphological changes before and after the test, providing further evidence of the excellent microstructural stability. In summary, the ZIS/SIS heterojunction exhibits outstanding photostability and durable cathodic protection performance in natural seawater, showing great potential for practical applications.

## 4. Discussion

**Figure 12:**
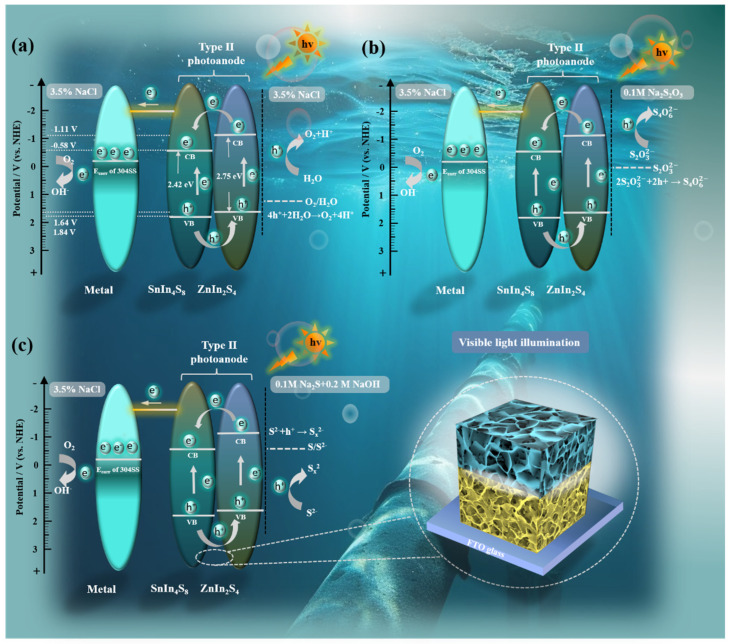
Proposed mechanisms of enhanced electron transfer in the PECCP performance of ZIS/SIS coupled with 304 SS in different solutions: (**a**) 3.5 wt% NaCl, (**b**) 0.1 M Na_2_S_2_O_3_, and (**c**) 0.1 M Na_2_S + 0.2 M NaOH.

Based on comprehensive experimental investigations, this study proposes a Type-II heterojunction-based PECCP mechanism for the ZIS/SIS heterojunction coupled with 304 SS, as illustrated in Figure 12. The heterojunction exhibits a unique structure-property relationship, featuring a stable 3D interlocked architecture composed of a bottom SnIn_4_S_8_ porous network and an upper layer of serrated ZnIn_2_S_4_ nanosheet arrays. Microstructural characterization reveals that ZnIn_2_S_4_ (hexagonal phase, a = 3.85 Å) and SnIn_4_S_8_ (cubic phase, a = 10.75 Å) form a 2:1 superlattice matching at the (111) plane (d = 7.60 Å), with an ultralow lattice mismatch of only 0.8%. This near-perfect lattice matching facilitates the formation of an atomically coherent interface [53], where [InS_6_] octahedra construct continuous charge transport pathways via sulfur-bridging bonds. The exceptional PECCP performance of the ZIS/SIS heterojunction stems from the synergistic effect between its electronic structure (Type-II band alignment) and its nanoarchitecture (3D interlocked network). The structural advantages of the material are manifested in three key aspects: (1) Type-II band alignment enables spatial separation of photogenerated electron-hole pairs; (2) the coherent interface significantly enhances the migration rate of photogenerated electrons; and (3) the 3D interlocked macroporous network (500–700 nm pore size) not only optimizes electrolyte diffusion and mass transport [35] but also establishes numerous mechanical anchoring points, enhancing structural integrity and ensuring sustained electron delivery pathways during long-term operation. These distinctive structural features endow the heterojunction with differentiated photoelectrochemical protective behaviors in various electrolyte systems, as detailed below.

When 3.5 wt% NaCl is employed as the electrolyte, under visible-light irradiation, as shown in Figure 12a, photogenerated electrons from the conduction band (CB) of ZnIn_2_S_4_ (E_CB_ = −1.11 V vs. NHE) are injected into the CB of SnIn_4_S_8_ (E_CB_ = −0.58 V vs. NHE) through a favorable energy gradient, followed by transfer to the metal surface via the FTO substrate. The migrated electrons react with dissolved oxygen to generate reactive intermediates such as hydroxide ions (OH^−^), thereby consuming corrosive species via electrochemical reduction and achieving metal protection. Concurrently, photogenerated holes from the valence band (VB) of SnIn_4_S_8_ migrate to the VB of ZnIn_2_S_4_. Due to the absence of effective hole scavengers in the NaCl electrolyte, holes accumulate on the ZnIn_2_S_4_ surface, inducing surface oxidation to form a passivation layer, which partially suppresses material self-corrosion.

In reductive electrolyte systems, the charge carrier transfer pathways undergo significant alterations. As illustrated in Figure 12b, in 0.1 M Na_2_S_2_O_3_ solution, under visible-light excitation, photogenerated electrons from the CB of ZnIn_2_S_4_ are transferred to the CB of SnIn_4_S_8_ via the Type-II heterojunction and subsequently migrate to the metallic cathode (304 SS) in the corrosion cell through an external circuit. Meanwhile, photogenerated holes from the VB of SnIn_4_S_8_ migrate to the VB of ZnIn_2_S_4_ and react with ${S_{2}O}_{3}^{2-}$ at the ZnIn_2_S_4_ surface, forming polythionates (${S_{4}O}_{6}^{2-}$, via $2{S_{2}O}_{3}^{2-}$ + 2h^+^ → ${S_{4}O}_{6}^{2-}$). This process not only promotes efficient charge separation but also mitigates photocorrosion of the photoanode by consuming holes. In the corrosion cell, the transferred electrons primarily participate in the oxygen reduction reaction at the 304 SS surface, where the stable Cr_2_O_3_ passive film significantly enhances electron transfer efficiency.

In a strongly reductive 0.1 M Na_2_S + 0.2 M NaOH electrolyte, the ZIS/SIS heterojunction demonstrates further improved charge separation and transfer efficiency. As shown in Figure 12c, Photogenerated electrons rapidly migrate to the metal surface via the heterostructure to participate in cathodic protection, while holes react with S^2−^ in the electrolyte to form polysulfides ($S_{x}^{2-}$). Notably, the generated polysulfides may establish a dynamic equilibrium (S^2−^ + h^+^ $⇌{\;S}_{x}^{2-}$) in the alkaline medium, maintaining S^2−^ concentration in the electrolyte and prolonging the protection duration [54]. Compared to other hole scavengers (e.g., ${S_{2}O}_{3}^{2-}$), Na_2_S exhibits superior performance due to its more negative oxidation potential (E = −0.508 V vs. NHE), enabling more efficient hole consumption without generating detrimental byproducts, thereby achieving optimal cathodic protection efficacy.

The outstanding performance of the ZIS/SIS heterojunction primarily stems from the enhanced charge separation efficiency and interfacial charge transfer capability enabled by the Type-II heterojunction. Across three different hole scavengers, the heterojunction effectively separates electron-hole pairs and suppresses metal corrosion through distinct reaction pathways. Particularly in the Na_2_S/NaOH electrolyte, the strong reducibility and alkaline environment further optimize charge carrier utilization, providing highly efficient and durable photoelectrochemical cathodic protection—especially for passivation-prone metals like 304 SS.

## 5. Conclusions

Based on the experimental results, the following conclusions are drawn:

(1) Synthesis and structural characterization (XRD, SEM, TEM, XPS): A three-dimensionally interlocked ZnIn_2_S_4_/SnIn_4_S_8_ (ZIS/SIS) Type-II heterojunction was successfully fabricated via a sequential solvothermal-hydrothermal method. XPS analysis confirms interfacial charge redistribution, with decreased electron density around Zn, In, and S in ZnIn_2_S_4_ and increased electron density around Sn in SnIn_4_S_8_, evidencing directional electron transfer from ZnIn_2_S_4_ to SnIn_4_S_8_.

(2) Optical and energy band properties (UV-vis DRS, Tauc plots, XPS valence band, Mott-Schottky): The heterojunction exhibits an intermediate bandgap of 2.60 eV, with its absorption edge lying between those of SnIn_4_S_8_ (560 nm) and ZnIn_2_S_4_ (500 nm). The Type-II band alignment creates a built-in electric field that promotes spatial charge separation.

(3) Photoelectrochemical cathodic protection performance (OCP, photocurrent density, i-V curves, EIS): The ZIS/SIS photoanode delivers stable photocurrent densities of 15.22 μA·cm^−2^ (3.5 wt% NaCl), 19.76 μA·cm^−2^ (0.1 M Na_2_S_2_O_3_), and 72.27 μA·cm^−2^ (0.1 M Na_2_S/NaOH). A maximum OCP shift of 720 mV is achieved in Na_2_S/NaOH. EIS reveals that the heterojunction has the smallest charge transfer resistance among the tested electrodes.

(4) Practical applicability and stability (long-term OCP test in natural seawater): In natural seawater (a scavenger-free, real marine environment), the heterojunction maintains effective cathodic protection for over 24 h, demonstrating excellent photostability and durability under practical conditions.

(5) Mechanistic understanding (comparative electrolyte study): The protection mechanism is electrolyte-dependent. In NaCl, electrons reduce dissolved oxygen; in Na_2_S_2_O_3_, holes are consumed by S_2_O_3_^2−^ oxidation; in Na_2_S/NaOH, a S^2−^/S_x_^2−^ dynamic equilibrium sustains hole scavenging. The low lattice mismatch (0.8%) and the 3D interlocked architecture synergize with the Type-II band alignment to enhance charge separation and transport.

While the ZIS/SIS heterojunction shows promising photoelectrochemical cathodic protection performance, several limitations must be addressed before realistic marine deployment. First, this study relies on indirect electrochemical indicators (OCP shifts and photocurrent density) without direct corrosion quantification (e.g., potentiodynamic polarization, corrosion current density, or mass loss). Second, no post-exposure surface characterization (SEM, profilometry) was performed to confirm the suppression of localized corrosion (pitting) on 304 SS. Third, the stability test was limited to 24 h under constant laboratory conditions; long-term durability under dynamic marine factors—such as wet/dry cycles, salinity fluctuations, temperature variations, and biofouling—remains untested. Fourth, dissolved oxygen concentration was not monitored, although oxygen reduction likely contributes to the cathodic protection. Fifth, key electrochemical analyses (EIS, Mott-Schottky) were performed in inert Na_2_SO_4_ rather than in the actual corrosive electrolytes, and no equivalent-circuit fitting was conducted. Finally, the observed XPS binding-energy shifts are small (0.1–0.2 eV) and were not verified by repeated measurements. To bridge the gap between laboratory findings and practical marine application, future work should include: (i) quantitative corrosion kinetics (Tafel plots, EIS on the protected metal, long-term immersion tests); (ii) surface analysis of the 304 SS after PECCP cycling (SEM, white-light interferometry, Raman spectroscopy); (iii) extended stability tests under cyclic marine conditions (wet/dry, salinity variation, temperature cycling, simulated biofouling); (iv) in situ monitoring of dissolved oxygen; (v) equivalent-circuit fitting of EIS and repeated XPS measurements with uncertainty analysis; and (vi) a prototype design with an external auxiliary unit circulating sacrificial electrolyte only on the photoanode side, leaving the metal side in natural seawater. Addressing these challenges will be the focus of our ongoing research.

## Data Availability

The original contributions presented in this study are included in the article. Further inquiries can be directed to the corresponding author.

## Abbreviations

The following abbreviations are used in this manuscript:

PECCP	photoelectrochemical cathodic protection
3D	three-dimensional
ZIS	ZnIn_2_S_4_
SIS	SnIn_4_S_8_
NSAs	nanosheet arrays
FTO	fluorine-doped tin oxide
TAA	thioacetamide
PTFE	polytetrafluoroethylene
OCP	Open Circuit Potential
WE	working electrode
SCE	saturated calomel electrode
RE	reference electrode
CE	counter electrode
VBM	valence band maximum
VB	valence band
NHE	normal hydrogen electrode
CB	conduction band
M-S	Mott-Schottky
